# Effect of *Saccharomyces boulardii* and Mode of Delivery on the Early Development of the Gut Microbial Community in Preterm Infants

**DOI:** 10.1371/journal.pone.0150306

**Published:** 2016-02-26

**Authors:** Natalia Zeber-Lubecka, Maria Kulecka, Filip Ambrozkiewicz, Agnieszka Paziewska, Milosz Lechowicz, Ewa Konopka, Urszula Majewska, Maria Borszewska-Kornacka, Michal Mikula, Bozena Cukrowska, Jerzy Ostrowski

**Affiliations:** 1 Department of Gastroenterology, Hepatology and Clinical Oncology, Medical Center for Postgraduate Education, Warsaw, Poland; 2 Neonatal and Intensive Care Department, Medical University of Warsaw, Warsaw, Poland; 3 Department of Pathology, The Children’s Memorial Health Institute, Warsaw, Poland; 4 Department of Genetics, Maria Sklodowska-Curie Memorial Cancer Center and Institute of Oncology, Warsaw, Poland; University of Illinois at Urbana-Champaign, UNITED STATES

## Abstract

**Background:**

Recent advances in culture-independent approaches have enabled insights into the diversity, complexity, and individual variability of gut microbial communities.

**Objectives:**

To examine the effect of oral administration of *Saccharomyces (S*.*) boulardii* and mode of delivery on the intestinal microbial community in preterm infants.

**Study Design:**

Stool samples were collected from preterm newborns randomly divided into two groups: a probiotic-receiving group (n = 18) or a placebo group (n = 21). Samples were collected before probiotic intake (day 0), and after 2 and 6 weeks of supplementation. The composition of colonizing bacteria was assessed by *16S ribosomal RNA* (rRNA) gene sequencing of fecal samples using the Ion 16S Metagenomics Kit and the Ion Torrent Personal Genome Machine platform.

**Results:**

A total of 11932257 reads were generated, and were clustered into 459, 187, and 176 operational taxonomic units at 0 days, 2 weeks, and 6 weeks, respectively. Of the 17 identified phyla, *Firmicutes Actinobacteria*, *Proteobacteria*, and *Bacteroidetes* were universal. The microbial community differed at day 0 compared with at 2 weeks and 6 weeks. There was a tendency for increased bacterial diversity at 2 weeks and 6 weeks compared with day 0, and infants with a gestational age of 31 weeks or higher presented increased bacterial diversity prior to *S*. *boulardii* administration. *Firmicutes* and *Proteobacteria* remained stable during the observation period, whereas *Actinobacteria* and *Bacteroidetes* increased in abundance, the latter particularly more sharply in vaginally delivered infants.

**Conclusion:**

While the mode of delivery may influence the development of a microbial community, this study had not enough power to detect statistical differences between cohorts supplemented with probiotics, and in a consequence, to speculate on *S*. *boulardii* effect on gut microbiome composition in preterm newborns.

## Introduction

The symbiotic relationship between gut commensal bacteria and underlying epithelial and lymphoid tissues results in both innate and adaptive immune defenses to pathogens and anoxious antigens, dietary nutrient and energy harvesting, and fermentation of non-digestible carbohydrates [1]. Microbial colonization of the gastrointestinal tract may have important consequences for human health and disease [2]. Although it is unknown how fecal microbes gain access to the uterus, the amniotic fluid is not sterile [3,4] and, therefore, initial microbial colonization can begin in utero as a part of in utero development [5,6]. The further development of infant gut microbiota is affected by various extrauterine factors, including the mode of delivery, gestational age, weight at birth, infant-feeding choice, and iatrogenic manipulations in the neonatal intensive care unit [7]. The normal colonization process is most likely to occur in full-term, vaginally delivered, and breastfed infants [1], while misdirected development of the gut bacterial community in preterm infants may affect the gut and immune function, leading to severe diseases such as necrotizing enterocolitis (NEC) [8–10]. Furthermore, the seeding and development of gut microbiota may have important pathophysiological consequences [9,11–14]. However, although infant gut microbiota possesses a relatively simple structure, its composition has been the subject of conflicting reports [15–18].

Probiotics are live microbes that exert beneficial effects on the host when administered in sufficient quantities. They exercise these effects by modulating the gut microbiota and promoting mucosal barrier functions and resistance to pathogens [19]. Administration of probiotics to pregnant and lactating women may be beneficial to allergy-prone infants born by cesarean section, and may result in reductions in the incidence of atopic dermatitis in infants with a maternal history of allergies [20,21]. In the neonatal period, probiotics are used to decrease the number of pathogenic enterobacteria in the gut, improve the general well-being of infants [22], and reduce the incidence of NEC [23–26]. However, the effect of probiotics on the incidence of NEC remains controversial [27]. While some studies have demonstrated that probiotics such as *Bifidobacterium* and *Lactobacillus* reduce the incidence of NEC in very low-birth-weight (VLBW) infants [28–30], others studies did not show such an effect [16,31,32].

*Saccharomyces boulardii* (*S*. *boulardii*) is a yeast-based probiotic that has been used for the treatment of gastrointestinal disorders associated with abnormal bacterial growth [22]. Although prophylactic *S*. *boulardii* supplementation is ineffective at reducing the incidence of death or NEC [27,33], and has no effect on D-xylose or fat absorption in VLBW [22], it improves feeding tolerance [22,34] and reduces the incidence of clinical sepsis, number of sepsis attacks, fungal colonization, and invasive fungal infection [27,34]. *S*. *boulardii* supplemented formula has been reported to have a beneficial effect on stool flora by bringing the GI flora composition closer to that of breastfed infants [22].

Recent advances in culture-independent approaches allow insights into the diversity, complexity, and individual variability of normal and probiotic-modified gut microbial community [14]. In this study, we assessed the short-term composition of colonizing bacteria by *16S ribosomal RNA* (rRNA) gene sequencing of extracted DNA from fecal samples to examine the development of the intestinal microbial community in preterm infants under oral administration of *S*. *boulardi* over a 6-week period.

## Material and Methods

### Ethics statement

The study conducted on infants was approved by the Medical University of Warsaw Ethics Committee (decision KB47/2012), and informed written consent was obtained from the parents of the infants. Gut microbiome study in adults was approved by the Cancer Center-Institute Ethics Committee (decision No. 70/2011), and informed written consent was obtained from all subjects. The study protocol conforms to the ethical guidelines of the 1975 Declaration of Helsinki.

### Studied subjects

In this double blind placebo controlled study, 55 preterm infants (31 males, 24 females), born between 25 and 33 weeks of gestation, were recruited from the Neonatal and Intensive Care Department of the Medical University of Warsaw, Poland. Infants were randomly divided into two groups. The study group (n = 28) received the commercially available *S*. *boulardii* (Dierol) at a daily dose of 2x10^9^ colony forming units, whereas the control group (n = 27) received a placebo (maltodextrin). Probiotics and placebo were administrated for 42 consecutive days (6 weeks). Administration of probiotics or placebo started when newborns could tolerate enteral nutrition after the removal of the umbilical vein or artery catheter, i.e. between 6 and 12 days of age. *S*. *boulardii* suspended in maltodextrin and maltodextrin alone (placebo) were provided in sachets (Sequoia, Warsaw, Poland) and were administered in a blinded manner. All products were stored at room temperature in closed sachets until use. The contents of the sachets were dissolved in 3 mL of feeding milk and administered either orally whenever possible or via an orogastric tube, followed by a 0.5 mL of flush with normal saline. Spontaneously produced fecal samples were collected by nurses or parents directly from the diaper into the collection tube using a sterile spatula. Samples were immediately frozen and stored at −20°C until processing. Stool samples were collected prior to supplementation with probiotics or placebo (0 days), and sample collection was repeated 2 and 6 weeks from the start of supplementation. All children finished the study, but a complete set of three stool samples was available from only 39 infants, of whom 18 and 21 received the probiotic and placebo, respectively. Only stool samples obtained from children who completed stool collection were used for microbiome analysis. All but two infants (one from each group) were fed with their mother’s breast milk throughout the study. As shown in Table 1, infants receiving *S*. *boulardii* did not differ from those receiving placebo. Stool samples were obtained also from 30 adults who were healthy hospital employees.

**Table 1:** Characteristics of the studied groups of infants.

	Probiotic group	Control group	P value
Total number	18	21	0.736
Female	7	8	0.452
Male	11	13	0.387
Cesarean section	11	16	0.136
Vaginal delivery	7	5	0.354
Gestational age* (weeks)	30.1±2.3	29.8±2.7	0.536
Weight* (grams)	1538±340	1503±379	0.742
Day of enrollment*	8±1.5	8±1.7	0.846

*The data are presented as means ± standard deviations. Statistical analyses were done using exact Fisher’s exact test, Yates correction, and the chi-square test.

### DNA extraction and metagenome sequencing

DNA isolation was performed using the QIAamp DNA Stool Kit protocol (Qiagen; Hilden, Germany) according to the manufacturer’s protocol. Briefly, a stool sample was scooped on dry ice, transferred to an Eppendorf tube, and weighted. Approximately 200 mg of stool sample was overlaid with 1 mL InhibitEX Buffer and vortexed thoroughly until homogenization was achieved. The sample was then heated at 95°C for 5 min followed by a centrifugation at 14 000 rpm for 2 min using a MiniSpin Plus centrifuge (Eppendorf; Hamburg, Germany) to pellet stool particles. A sample of the supernatant (200 μL) was transferred into a fresh tube, mixed with 15 μL Proteinase K and 200 μL of AL buffer, and incubated at 70°C for 10 min. Two hundred μL of ethanol was added and DNA was recovered on a QIAamp spin column according to the QIAamp DNA Stool Kit protocol. DNA was eluted then measured with Nanodrop (Thermo-Fisher; Waltham, MA, USA) spectrophotometer and stored in EB buffer at –20°C until further analysis.

#### Bacterial DNA detection and quantification

For bacterial DNA quantification, the PCR-based Femto Bacterial DNA Quantification Kit (Zymo; Irvine, CA, USA) was used. Quantitative PCR (qPCR) reactions were run in triplicate on the 7900HT Real-Time PCR System (Life Technologies; Carlsbad, CA, USA) using the thermocycling parameters and reaction volumes recommended in the manufacturer’s protocol (Zymo; Irvine, CA, USA). DNA standards provided in the kit (*E*. *coli* strain JM109) were used to generate a standard curve to quantify the amount of bacterial DNA in the samples.

#### 16S rRNA sequencing

Sequencing was performed using Ion 16S Metagenomics Kit (Life Technologies; Carlsbad, CA, USA) on the Ion Torrent Personal Genome Machine (PGM) platform (Life Technologies; Carlsbad, CA, USA). Briefly, 10ng of DNA was subjected to amplification of 16S rRNA libraries. Primers were partially digested and adapters ligated to the amplicons, purified using the Agencourt AMPure XP beads (Beckman Coulter; Pasadena, CA, USA) according to the manufacturer’s protocol, and stored at -20°C until further processing. The concentration of each 16S library was determined by qPCR using the Ion Library Quantitation Kit and a Qubit fluorometer (Life Technologies; Carlsbad, CA, USA). The library was diluted to ~100 pM prior to template preparation. Template preparation of the barcoded libraries was performed using the Ion PGM Template OT2 400 Kit (Life Technologies; Carlsbad, CA, USA) and the Ion OneTouch 2 System (Life Technologies; Carlsbad, CA, USA). A maximum of 32 barcoded 16S samples were sequenced on a Ion 318 v2 chip (Life Technologies; Carlsbad, CA, USA) using the Ion PGM Sequencing 400 Kit (Life Technologies; Carlsbad, CA, USA) according to the manufacturer’s instructions. Sequencing data in BAM format have been deposited at The European Bioinformatics Institute (EBI) Metagenomics repository under accession number PRJEB9898. Mock community dataset was generated from a mixed bacteria genomic DNA (HM-276D, BEI resources) and is available under https://ioncommunity.thermofisher.com/docs/DOC-8424 after free registration.

#### Bacterial taxonomy identification

Unmapped BAM files were converted to FastQ files using Picard’s SamToFastq [35]. Sequences were filtered according to their quality using the FastQ quality filter in the FASTX-Toolkit [36], resulting in more than 80% of the filtered sequences’ bases having a quality of 20 or higher. Further steps in the analysis were performed using the software Mothur [37]. FastQ files were converted to FASTA format. Only sequences with lengths between 200 and 300 bases were kept for further analysis. Chimeric sequences were identified using Uchime [38], which was run with standard parameters and our sequence collection as the database. Sequences marked as chimeric were then removed. The remaining 16S rRNA sequences were classified using the Wang method and the SILVA bacterial 16S rRNA database as a template (release 102, [37]), at a bootstrap cutoff of 80%. The taxonomic profile was created using a modified script from STAMP [39].

#### Data visualization and statistical analyses

Data visualization, including the percentage of bacterial taxa in each sample, statistical analyses, and principal component analysis (PCA) were performed using R and the graphics package ggplot2 [40]. The phylogenetic tree was prepared in MEGAN5 [41]. The circular visualization of OTUs present at each time-point was prepared in GraPhlAn [42]. Differences between groups in relative bacteria DNA amounts were evaluated by the Mann-Whitney U-test. For statistical analyses of taxonomy, relative abundances of each OTU were computed: the number of sequences assigned to a given OTU was divided by the total number of good quality sequences in the sample. Differences in first and second principal component between two groups (*S*. *boulardii* versus placebo and Cesarean section versus vaginal delivery) were determined with Student’s t-test. Differences between time points were determined using repeated measures ANOVA and Student’s pairwise t-test as a post-hoc test. Taxonomic contrasts between two groups (*S*. *boulardii* versus placebo and Cesarean section versus vaginal delivery) were determined by the Mann-Whitney U-test. Differences between time points were determined with the Mann-Whitney paired U-test. Gut microbiota with nearly constant relative abundance at operational taxonomic unit (OTU) levels (IQR (abundance) <0.5) were removed from taxonomic analyses. P-values were corrected for multiple hypothesis testing using the Benjamini–Hochberg procedure to control the false discovery rate (FDR) [43]. Species community α-diversity expressed by Simpson’s Diversity index was calculated in Mothur. Contrasts between two groups (*S*. *boulardii* versus placebo and Cesarean section versus vaginal delivery) were determined by Mann-Whitney U-test. Differences between time points were determined using repeated measures ANOVA and pairwise Student’s t-test as post-hoc test. Additionally, a linear model was performed at genus level, with log 10-transformed relative abundances as the response variable and *S*.*boulardii* intake, mode of delivery, and time as predictors. P-values associated with predictors were also corrected for multiple hypothesis testing using the Benjamini–Hochberg procedure to control the FDR. The power analysis was conducted in GPower 3.1 [44]. The total bias was calculated as described by Brooks et al. [45]. Briefly, if *A* was the mixing ratio of a given bacterium in a mock community dataset and *B* was the observed proportion, the bias was the difference *A−B*. A negative value denoted that the bacterial signal was suppressed, while a positive value denoted that the signal was amplified.

## Results

### Ion 16S Metagenomics Kit validation

It becomes more appreciated that portraying gut microbiota composition, also in infants, with 16S rRNA gene sequencing of hypervariable regions can be influenced by preanalytical variables including the sample processing and choice of PCR primers [46]. Therefore, we conducted a validation of the 16S Metagenomics Kit, that allows a consensus view across 6 regions (V2, V3, V4, V6-7, V8 and V9), by comparing its qualitative and quantitative performance to already published dataset of a synthetic 20-organism mock bacterial community that was generated on Illumina's sequencing platform with a use of V4 and V4-5 amplicon assays [47]. All the assays properly identified the composition of a community. For a quantitative comparison we calculated the total bias [45] by comparing the results of a given assay with the prescribed mixing ratio in a mock community. This comparison showed that 16s Kit assay had the lowest median bias (S1 Fig) indicating that it reliably captures and quantifies taxonomic composition of a reference mock community.

### Overall characteristics of the gut microbiome in premature infants

**Fig 1:**
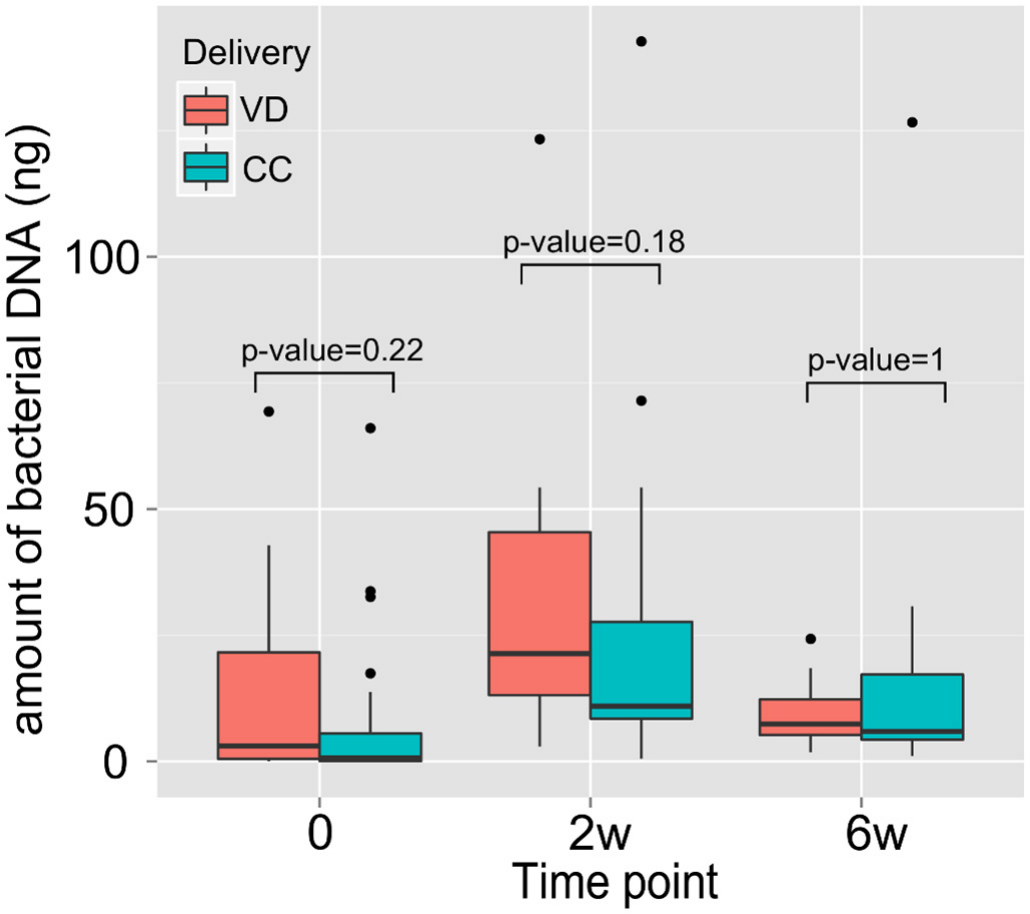
Relative levels of bacterial DNA extracted from fecal samples in preterm infants delivered vaginally (VD) or by cesarean section (CS). 0, day 0; 2w, 2 weeks; and 6w, 6 weeks after supplementation.

Fecal samples were collected 6–12 days following birth, which was prior to supplementation with *S*. *boulardii* or placebo (day 0) and 2 weeks and 6 weeks after supplementation. Total bacterial DNA from the fecal samples was analyzed by qPCR. Relative amounts of bacterial DNA did not reveal any statistically significant differences at any time point (p-value in Mann-Whitney U test > 0.05) (Fig 1).

The distribution of bacterial groups in the gut microbial community was analyzed by sequencing of the 16S rRNA hypervariable regions of bacterial DNA on the PGM platform. On average, 101985 sequences that passed all the quality filters were generated per library, and a total of 11932257 good quality sequence reads were generated and mapped to Bacteria and Archaea in the SILVA database. The sequences were sorted into 459, 187, and 176 OTUs using SILVA taxonomy at day 0 and 2 and 6 weeks after supplementation, respectively, and of these, 49, 46, and 50, respectively, were represented by more than 0.01% of the reads.

**Fig 2:**
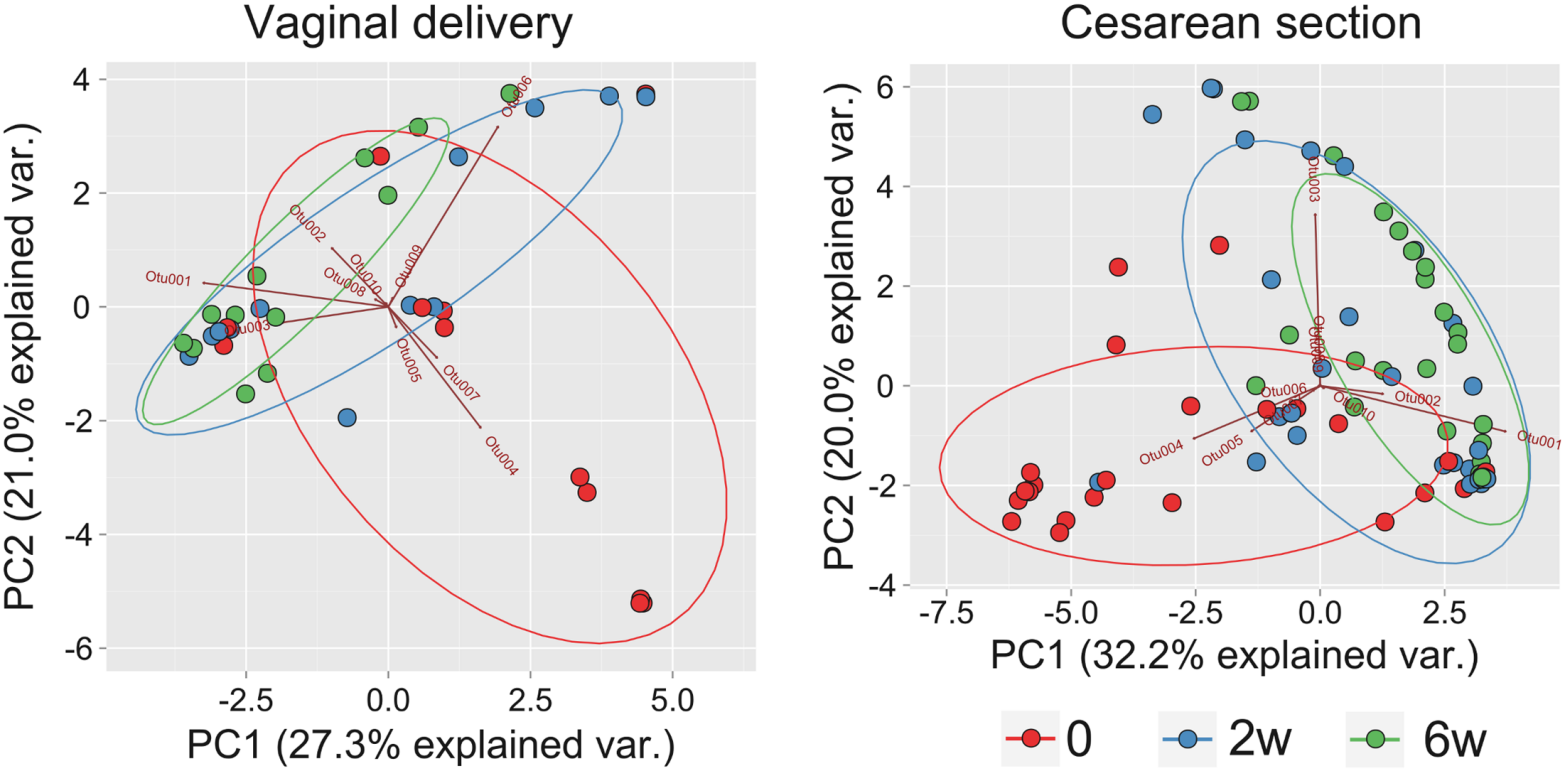
Results of PCA analysis of infant gut microbiome in infants delivered vaginally compared with those of infants delivered by cesarean section. Only 10 of the most abundant OTUs are shown. PC, principal component; var., variance; 0, day 0; 2w, 2 weeks; and 6w, 6 weeks after probiotic or placebo supplementation.

PCA based on the OTUs showed that the infant microbiome at day 0 differed to that at 2 weeks and 6 weeks following supplementation (Fig 2). The differences in the first and second principal component were statistically significant in each group: p-values in repeated measures ANOVA were 0.01 and 0.004 for vaginally delivered infants and 7.02e-08 and 2e-05 for infants delivered by Cesarean section.

Of the 17 bacterial phyla identified at the early phase of gut microbiome development in preterm infants, three phyla (*Firmicutes*, *Actinobacteria* and *Proteobacteria*) exhibited 100% prevalence, while *Bacteroidetes* was present in 97%, 89%, and 97% of samples collected at day 0, and 2 and 6 weeks after supplementation, respectively. *Cyanobacteria* were found in 60%, 49%, and 43% of samples collected at day 0, and 2 and 6 weeks after supplementation, respectively, and *Tenericutes* was found in almost 40% of samples at each time point. An additional five phyla (*Verrucomicrobia*, *Acidobacteria*, *Fusobacteria*, *Planctomycetes*, and *Tenericutes*) were detected in more than 10% of samples, primarily on day 0.

**Fig 3:**
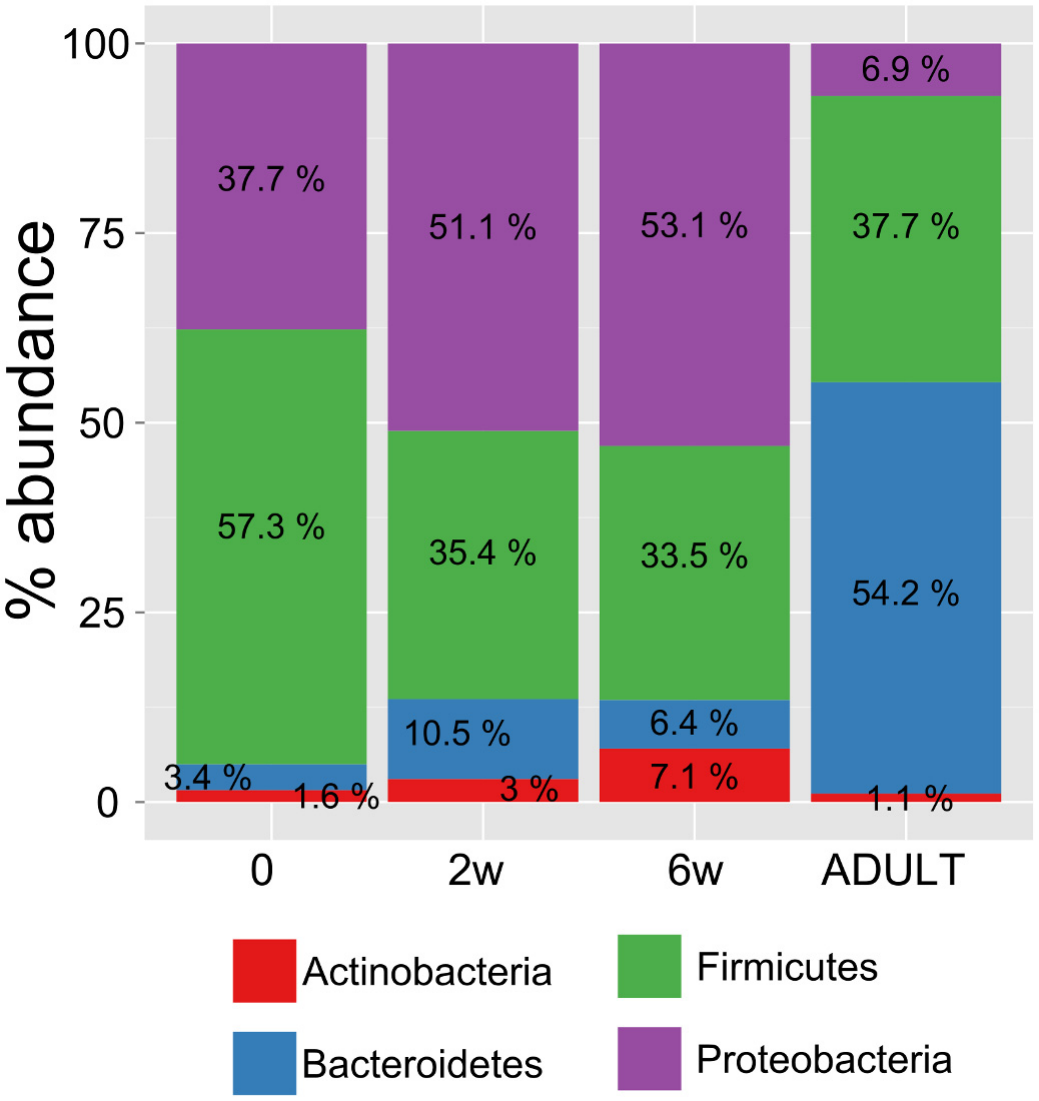
Level of abundance of most prevalent phyla in stool samples of preterm infants collected at various time points. Samples were collected 6–12 days following birth (0w or day 0 prior to supplementation), and 2 weeks (2w), and 6 (6w) weeks after probiotic or placebo supplementation, and from healthy adults (ADULT).

*Firmicutes*, *Actinobacteria*, *Proteobacteria*, and *Bacteroidetes* represented the bacterial core set comprising of 99.9% of the sequences, whereas the remaining 0.1% of sequences comprised rare bacterial phyla. Although the dominant bacterial phyla were identical in both the immature and mature gut microbial community, differences in the microbial composition of preterm infants and adults were visible (Fig 3). *Proteobacteria* was predominant in healthy adults compared with preterm infants at each time point, whereas *Bacteroidetes* was abundant in healthy adults compared with preterm infants at each time point.

**Fig 4:**
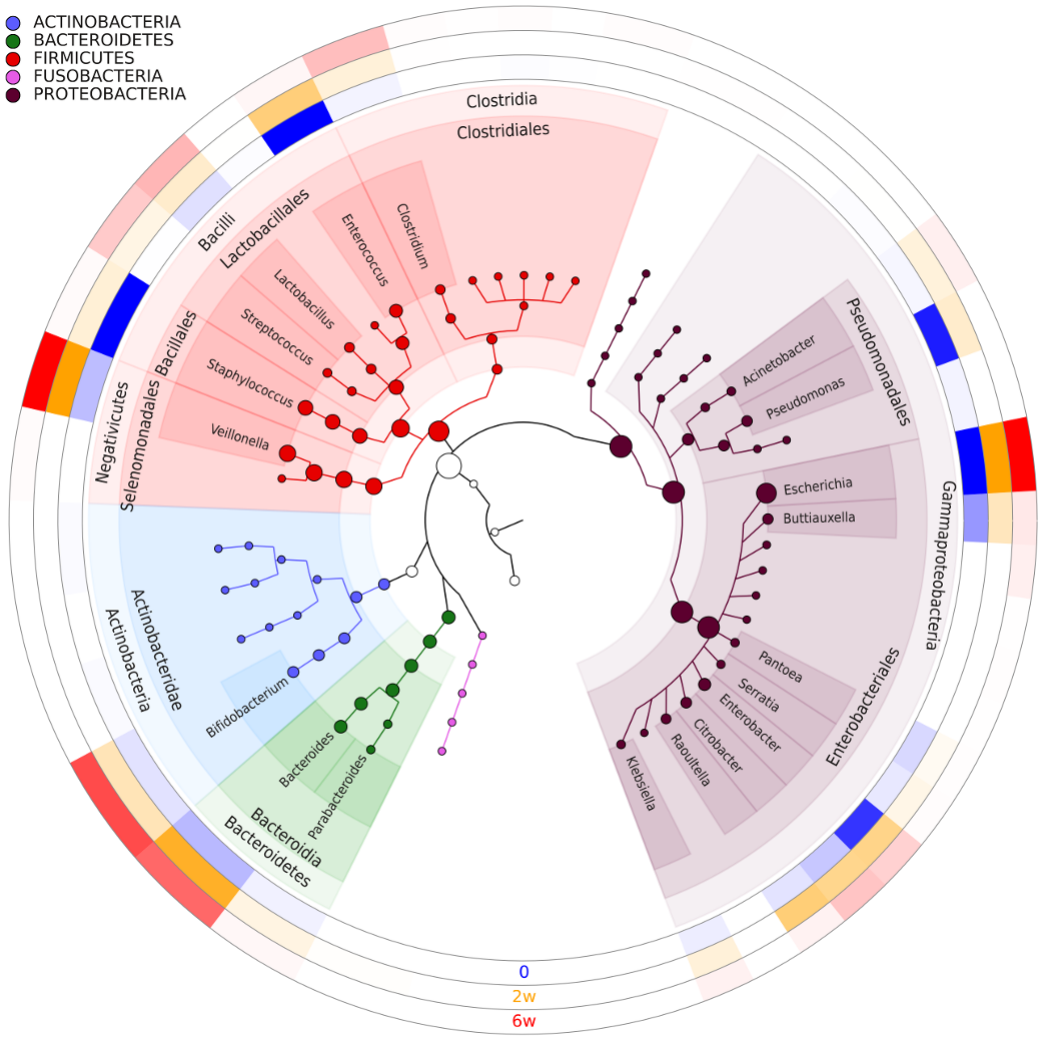
Composition of the gut microbiome at the early phase of development dominated by four phyla: *Actinobacteria*, *Bacteroidetes*, *Firmicutes*, and *Proteobacteria*. A phylogenetic tree demonstrating bacterial abundance is present in the center. Each circle depicts a heatmap, representing a one-time probe. The more intense the color on the heatmap, is, the larger the percentage of reads from a given genus is.

An overview of 39 genera that were present in more than 1% of reads is depicted in Fig 4. *Escherichia* was present in preterm infants regardless of fecal sampling time and mode of delivery. The most notable changes over time were the disappearance of *Staphylococcus* and *Pseudomonas* 2 weeks following commencement of probiotic intake (paired Wilcox test between time 0 and 2 weeks, P = 0.002 and P = 0.008, respectively), a decrease in *Enterococcus* (paired Wilcox test between time 0 and 6 weeks, P = 0.004), and an emergence of *Veilonella* (P = 0.0002), *Clostridium* (P = 0.02), and *Bifidobacterium* (P = 0.0002) 6 weeks after supplementation, regardless of the mode of delivery.

**Fig 5:**
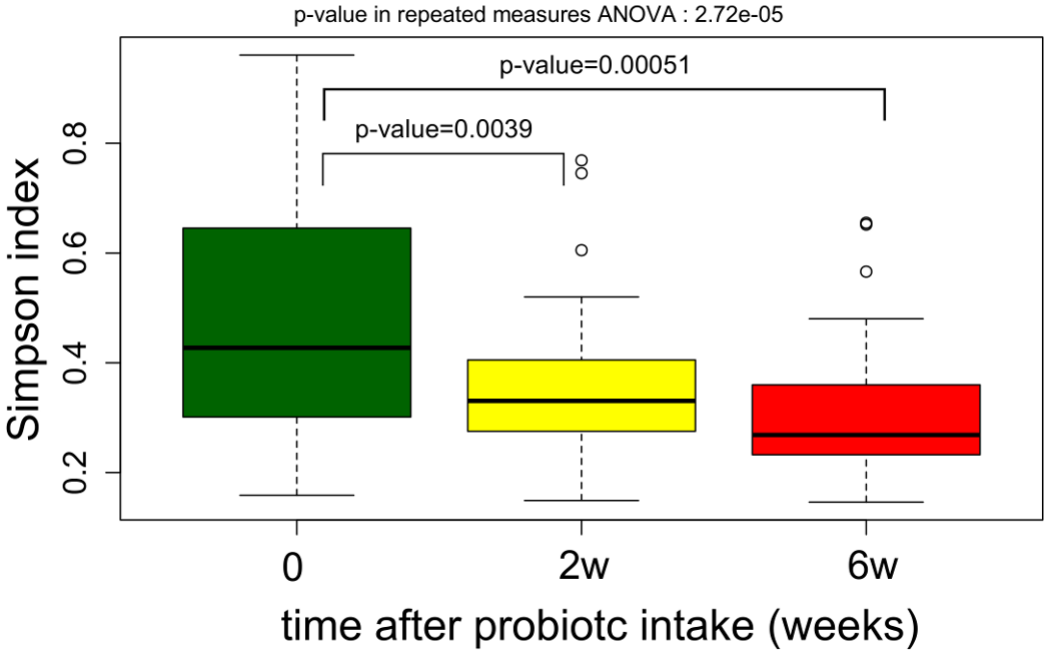
Boxplot of community α diversity expressed by the Simpson index in stool samples collected from preterm infants at day 0 (prior to supplementation), and 2 (2w) and 6 (6w) weeks following probiotic or placebo supplementation. P-values on the figure are p-values obtained by the pairwise t-test, adjusted for multiple testing by the Benjamini–Hochberg procedure.

**Fig 6:**
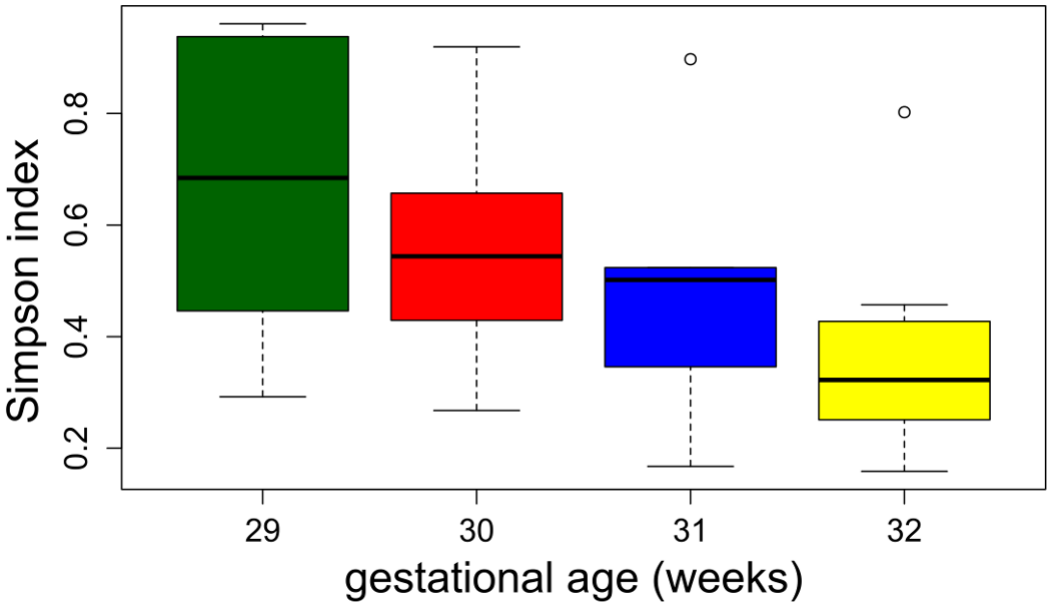
Boxplot of community α diversity at day 0 (prior to supplementation) according to the Simpson index and categorized according to gestational age at birth. Data are shown only for the weeks in which at least five infants were born.

The increase in α-diversity as a function of time (Fig 5) revealed that the bacterial community became progressively more complex at 2 and 6 weeks after supplementation compared with the bacterial community at day 0. This difference was statistically significant with a p-value of 2.72e-05 in repeated measures ANOVA. A tendency for intestinal microbial complexity to increase was observed in infants born after 31 weeks of gestation (Student’s t-test between children with gestational age lower than 31 weeks and children with gestational age greater than 30 weeks; P = 0.0249) (Fig 6).

### S. *boulardii* supplementation does not influence the early gut microbial community in preterm infants

**Fig 7:**
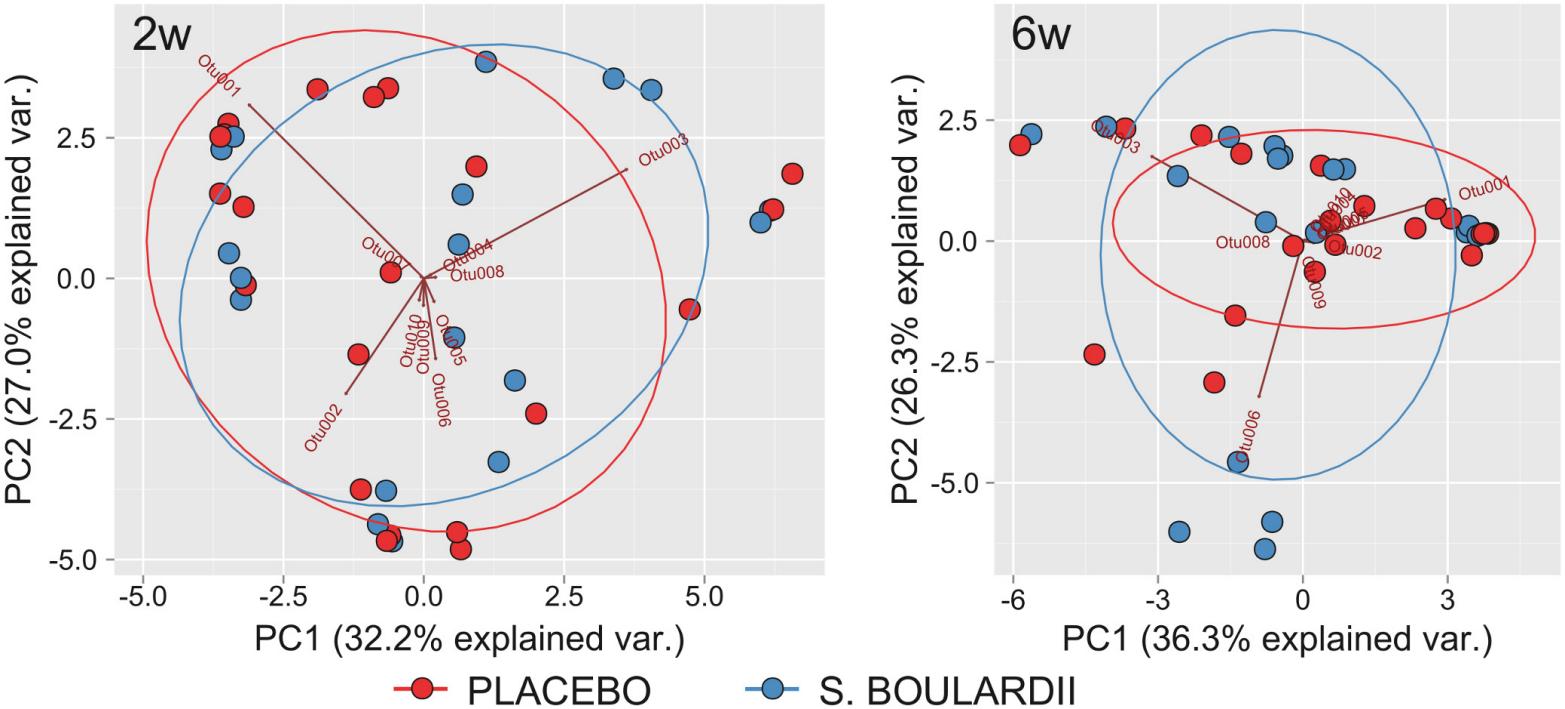
PCA analysis of the preterm infant gut microbiome grouped according to the administration of *Saccharomyces boulardii* or placebo, categorized according to the time points 2 (2w) and 6 (6w) weeks following supplementation. Only ten of the most abundant OTUs are shown. PC, principal component; var., variation; and *S*. *Boulardii*, *Saccharomyces boulardii*.

**Fig 8:**
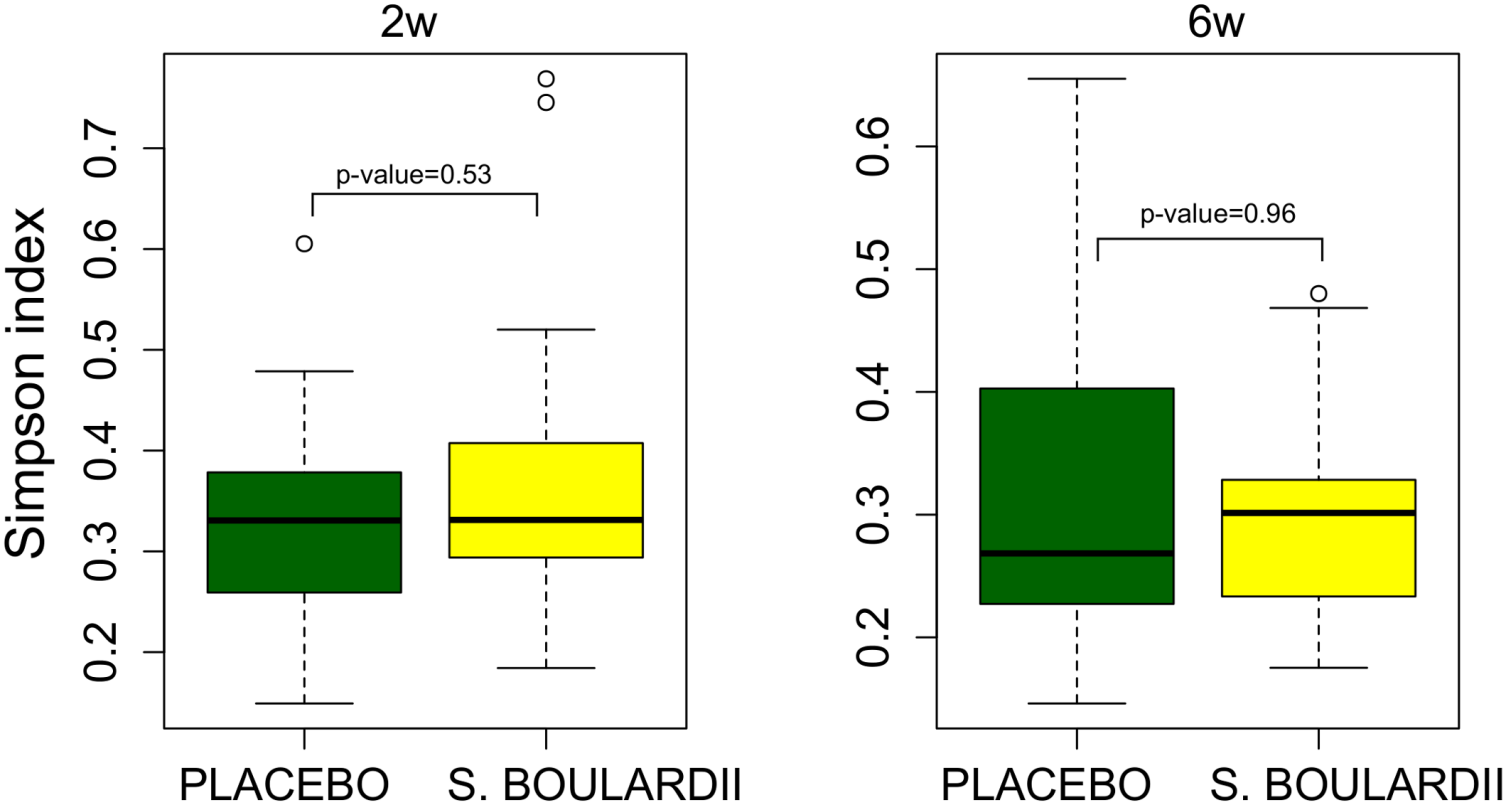
Boxplot of community α diversity, as expressed by the Simpson index, categorized according to the administration of the probiotic *Saccharomyces boulardii* (*S*. *Boulardii*) or placebo at 2 (2w) and 6 (6w) weeks following intervention. P-values in Mann-Whitney U-test are shown for respective times.

Although PCA visualization based on the relative abundance of OTUs revealed a cluster of four samples from children supplemented with *S*. *boulardii* (Fig 7), the differences in the first and the second principal components were not statistically significant for either 2 weeks (p-values in Student’s t-test; 0.505 and 0.91 for principal component 1 and 2, respectively) or 6 weeks (p-values are 0.27 and 0.508, respectively) after the probiotic administration. The administration of probiotics did not alter also the microbial α-diversity (Fig 8) or the microbiota OTUs at any taxonomical level.

### Effect of mode of delivery on the gut microbial community of preterm infants

**Fig 9:**
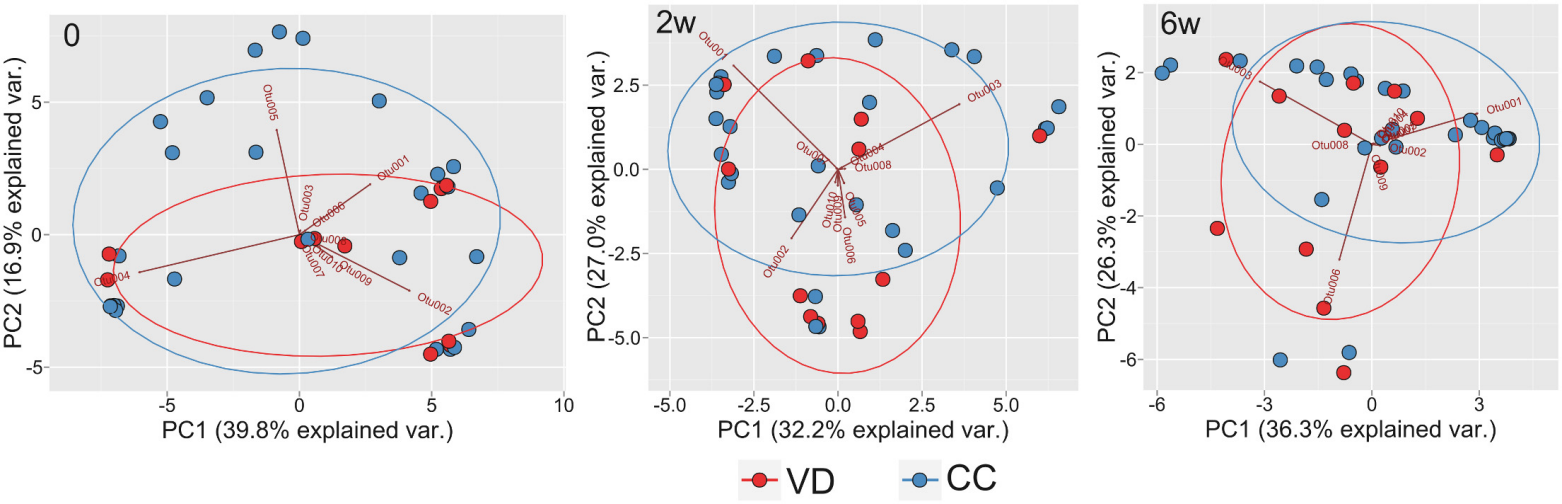
PCA analysis of the infant gut microbiome, categorized according to time points and mode of delivery. Only the ten most abundant OTUs are shown. 0, prior to supplementation; 2w and 6w, 2 weeks and 6 weeks following commencement of supplementation; PC, principal component; var., variation; VD, vaginal delivery; and CC, cesarean section.

The mode of delivery has been found to strongly affect neonatal microbiome species[6,20]. In our study, although clustering patterns for the relative abundance of OTUs showed some visible differences between the gut microbiome of infants delivered vaginally and those of infants delivered by cesarean section at each time point (Fig 9), overall differences between groups in the first and the second principal component were not statistically significant: p-values for consecutive time points in Student’s t-test were 0.411, 0.983, 0.414 for the first principal component and 0.101, 0.0678, 0.2302 for the second, respectively.

The genera from phylum *Bacteroidetes*: *Bacteroides* and *Parabacteroides* were almost exclusively present in vaginally delivered infants at day 0: 8% and 2% sequences respectively versus 0.6% and 0.02% sequences for infants delivered via Cesarean section and 2 weeks after supplementation (21% and 2% sequences for vaginally delivered infants versus 2% and 0.001% sequences for infants delivered via Cesarean section). Mode of delivery is a significant predictor for relative abundance of both this genera, with an adjusted p-value of 0.0102 and 7.34e-05, respectively.

## Discussion

By 12–18 months of age, an infant’s intestine is colonized by more than 1,000 separate bacterial species [15]. The maturation of the gut microbiota is a non-random process, and is influenced by dietary and medical factors [48,49]. Metagenomic shotgun sequencing on fecal samples from full-term infants and their mothers demonstrated that mode of delivery and cessation of breastfeeding were two key factors driving the assembly of an adult-like gut microbiota during the first year of life [49], whereas in the study by La Rosa [48], gestational age at birth was implicated as the main driver of gut microbiota maturation in preterm neonates. Over time, microbiota maturation leads to more complex and less dissimilar gut microbial configurations. In the study by Abrahamsson and colleagues [50], a low total diversity of gut microbiota during the first month of life was associated with asthma in children at 7 years of age; therefore, factors affecting infant microbial colonization after birth may impact the development of allergies in childhood. There is a growing consensus that the functional state of the gut microbiota is more dependent on the diversity of species (functional diversity) than on taxonomic diversity, and alterations of functional diversity may be related to human health problems [51]. Our study revealed a relation between gestational age and the complexity of gut microbiota, as an earlier gestational age was associated with a less complex gut microbiota approximately 1 week following birth (day 0; Fig 6).

Although no sequencing technique is considered a gold standard for the study of microbiome taxonomic diversity, two methods, sequencing the variable regions of 16S rRNA gene and shot-gun sequencing of total microbial genome mixtures, are considered appropriate for the exploration of microbial communities. Both of them are taxonomically and phylogenetically informative and both present unique analytical challenges, including data visualization and statistical testing [52,53].

Consistent with previous studies (reviewed in [6]), the early gut bacterial colonizers in our group of preterm infants belonged to four dominant phyla, *Firmicutes*, *Proteobacteria*, *Actinobacteria*, and *Bacteroidetes*, which comprised 99.9% of the sequences. Of these, *Firmicutes* and *Proteobacteria* represented 94% of the sequences. These two phyla tended to be stable during the first 6 weeks of life.

By contrast to previous observations in full term infants [54], the 16S rRNA profiling results of a previous study by Arboleya et al. and our study indicate that the delivery mode has only a limited effect on gut microbiota composition in preterm infants [55]. The only difference observed in our study suggested that the mode of delivery was a predictor of *Bacteroides* and *Parabacteroides* genera abundance, and Jakobsson et al. [56] found a delayed colonization by *Bacteroides* during the first days of life in cesarean delivered newborns. On the other hand, they reported a lower percentage of members of the family *Bacteroidaceae* during the first months of life in preterm infants than in full term infants [55].

*Bacteroides* plays an important role in activating the immature immune system to anti-allergic responses. In infants and young children suffering from a food allergy or atopic dermatitis, the first symptoms of allergy are associated with a decreased abundance of *Bacteroides* [57], and newborns with lower numbers of *Bacteroides* have a higher risk of allergic diseases [58]. Several studies have shown that cesarean section is a risk factor for future allergies [59].

Previously, a number of studies have analyzed whether probiotics have beneficial effects in preterm newborns, with the aim of preventing nosocomial infections and NEC [60]. The primary risk factors for NEC are prematurity and altered bacterial colonization after initiation of enteral feeding [8]. Samples from patients with NEC show less microbial diversity than that of preterm infants, and have an increased abundance of *Gammaproteobacteria*, whereas in infants who go on to develop NEC, a significant decrease in the phylum *Firmicutes* is observed [8]. Furthermore, in twins, of whom one developed NEC and the other did not, a clear separation in functional gene sets prior to the onset of NEC was identified [8]. Therefore, the hypothesis that the protective effect of probiotics against NEC may result from the early modification of gut microbiota, resulting in enhanced maturation of the intestinal barrier functions and/or immune responsiveness, appears plausible.

Costalos and co-workers, [22] used culture-based techniques to demonstrate a significant decrease in *Escherichia coli* and *Enterococci* and an increase in *Bifidobacteria* and *Staphylococci* in stool samples from preterm infants supplemented for 30 days with *S*. *boulardii*. By contrast, our study provides no evidence for a probiotic effect of *S*. *boulardii* on the development of the early gut microbial community in preterm infants. Since there are evident methodological differences between both studies, these differences suggest inconsistent results. While culture-based techniques allow for the identification of only a small proportion of gut microbial colonizers, bacterial taxonomic classification of 16S rRNA gene sequences (culture-independent technique) provides a comprehensive perspective of the microbial configuration in which low abundant colonizers may be overshadowed by more dominant ones. If this is the case, the lack of statistical significance in the taxonomic differences between infants supplemented with probiotics and the placebo group may reflect the weak impact of *S*. *boulardii* on gut microbiome development in premature newborns.

In infants supplemented with *S*. *boulardii*, the standard deviation in the relative abundances of OTUs ranged from 70% to 412% of the mean value, with a very uneven level of taxonomic coverage, indicating high inter-individual variability in preterm infants. These observations are in agreement with those of previous reports [55,61–63]. To obtain a statistically significant result in Mann-Whitney U-test with power of 80% in a comparison between two groups for a moderate size effect (that is a Cohen’s d effect of 0.5), it would have been necessary to include at least 67 children in the group; however, this was not possible in our study. On the other hand, the effect size in Mann-Whitney paired U-test with power of 80% and 39 samples is considerably smaller and reaches 0.47. Therefore, our study was better suited to discovering differences between time points rather than between groups. We employed various linear models as an alternative method of analysis; however, the distributions of the relative abundances of OTUs in the NGS-derived metagenomic dataset were far from normal [64] and, therefore, the applicability of these models was also limited.

Although the development of distinctive microbial communities has been previously observed in NEC patients, it remains unknown whether this is the result of the presence of pathogenic bacteria, the absence of beneficial bacteria, or a combination of both [8]. In this study, NEC was not diagnosed in newborns, and thus, this study did not analyze the microbial configuration that precedes disease development. The positive effect of *S*. *boulardii* supplementation may not solely be the result of the modulation of intestinal microbiota, but may also result from the production of polyamines, which are essential for cell growth and differentiation [65]. Thus, in line with previous studies [22,33,34], *S*. *boulardii* was well-tolerated by preterm newborns in this study; however, it was not possible to test its ability to prevent NEC.

In conclusion, our study has confirmed that the mode of delivery may influence the development of a microbial community, but had not enough power to detect statistical differences between cohorts supplemented with probiotics, and in a consequence, to speculate on *S*. *boulardi* effect on gut microbiome composition in preterm newborns.

## Supporting Information

S1 Fig Observed bias by assay for a mock community. Bias values were expressed as a difference between prescribed and observed ratio for a given bacterium. *Single 1*. and *single 2*. refer to assays with a single-end sequencing targeting region V4, the *paired* is an assay with a pair-end sequencing of V4-V5 amplicon. *16s Kit* represents the results for a sequencing protocol used in this study.
(TIF)
