# Supplementary figures and images for: Effect of Saccharomyces boulardii and Mode of Delivery on the Early Development of the Gut Microbial Community in Preterm Infants
Source: PLoS One. 2016 Feb 26;11(2):e0150306. doi: 10.1371/journal.pone.0150306 (PMC4769247; doi:10.1371/journal.pone.0150306)

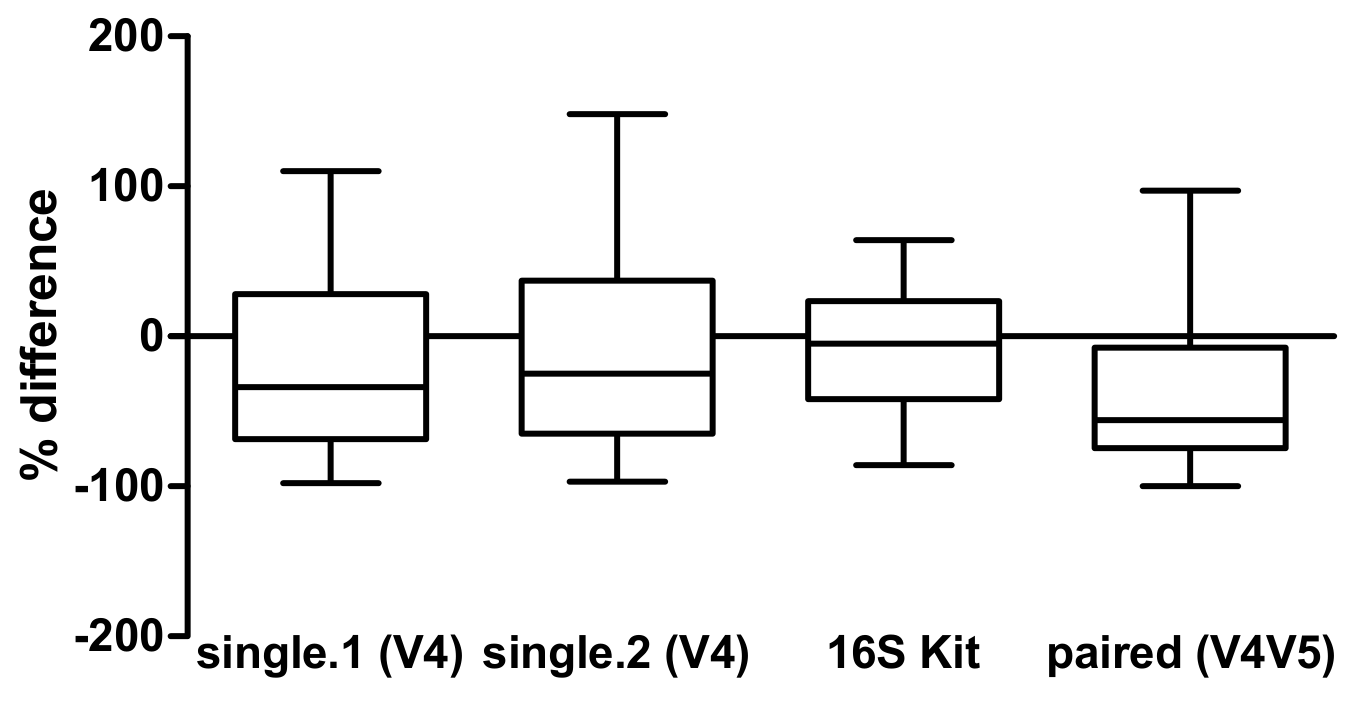

Supplement: S1 Fig — Bias values were expressed as a difference between prescribed and observed ratio for a given bacterium. Single 1. and single 2. refer to assays with a single-end sequencing targeting region V4, the paired is an assay with a pair-end sequencing of V4-V5 amplicon. 16s Kit represents the results for a sequencing protocol used in this study. (TIF) [file pone.0150306.s001.tif]
